# Dysbiosis in salivary bacterial diversity of postpartum females and its association with oral health problems and APOs

**DOI:** 10.1016/j.crmicr.2021.100032

**Published:** 2021-04-20

**Authors:** Bibi Khadija, Lal Badshah, Ayesha Siddiqa, Bushra Rehman, Samiaa Anjum, Anum Saeed, Shahzad Hussain, Rani Faryal

**Affiliations:** a Department of Microbiology, Faculty of Biological Sciences, Quaid-i-Azam University, Islamabad, Pakistan; b Department of Medical Laboratory Technology, University of Haripur, Haripur, KPK, Pakistan; c Drug Control and Traditional Medicine Department, National Institute of Health (NIH), Islamabad, Pakistan

**Keywords:** Adverse pregnancy outcomes, Dental caries, Gingivitis, Microbiome, Postpartum females

## Abstract

The present study investigates the dysbiosis in salivary bacterial diversity by culture-dependent and independent methods. Culturable aerobic and facultative anaerobic bacterial diversity was studied in saliva collected from 267 postpartum and 54 nonpregnant females by using standard microbiological methods. For unculturable bacterial diversity, DNA from saliva samples of four selected females was sequenced by targeting V4 region of 16S rRNA. In postpartum females, *S. mutans* was significantly more prevalent. Its colonization was also seen significant among females having gingivitis (*P* < 0.01), dental caries (*P* < 0.01), and in those giving birth to low weight baby. In postpartum group, 65.16% females were culture positive for *Staphylococcus*, 12.73% Gram positive rods, 10.48% *N. meningitides*, 6.36% *K. pneumoniae*, 5.61% *Enterobacter* species and 2.62% *E. coli*. Isolates showed high biofilm forming ability and antibiotic resistance. Upon analysis of unculturable bacterial diversity, a total of 16 phyla and 156 genera were observed. Alpha diversity was decrease in postpartum female having oral health issues with pre-term low weight birth, compared to females with full term birth. Bray-Curtis dissimilarity was highest between female with dental issues and different pregnancy outcomes. Bacterial diversity and abundance altered among females with different oral health conditions and pregnancy outcomes, and also have pathogenic potential.

## Highlights

• In present study, salivary microbial diversity of postpartum females was compared with nonpregnant females.
• Microbial colonization was raised in postpartum females especially those having oral health issues.
• Isolated bacterial species showed enhanced biofilm forming and multi drug resistance.
• Alpha diversity was decrease in postpartum female having oral health issues with PLWB.
• Bray-Curtis dissimilarity was highest between females with dental issues and different pregnancy outcomes.

## 1 Introduction

Human oral cavity harbours unique microbial ecosystem, made up of over 700 distinct species of bacteria (Cobb et al., 2017; Digiulio et al., 2015). Balance in this microbial ecosystem is important for maintaining local and systematic health of an individual (Verma et al., 2018). However, any alteration in these health associated communities can create dysbiosis, leading to the development of oral disorders such as periodontal disorders and caries lesions (Adams et al., 2017; Cobb et al., 2017). Oral microbes specifically colonize different ecological niche (Dewhirst et al., 2010). By using next generation sequencing (NGS) of 16S rRNA gene from nine different sites in oral cavity of over 200 healthy individual, Human Microbiome Project (HMP) assessed that these sites are colonized by 13–19 different bacterial phyla and 185–322 genera (Zaura et al., 2014).

Salivary microbiota comprised of bacteria shed from different oral surfaces (Belstrøm et al., 2017; Sampaio-Maia et al., 2016). Although different individual shared similar salivary microflora, there is an inter-individual differences (Pelzer et al., 2017; Sampaio-Maia et al., 2016). Composition of salivary flora also vary in patients with different disorders like periodontitis, dental caries, oral squamous cell carcinoma and pancreatic cancer as compared to healthy population, which suggesting the possible use of salivary microbiota as biomarkers for disease diagnosis (He et al., 2014).

Beside other conditions, pregnancy is one of the state during which almost every part of female body is disturbed including oral health. Oral microbial diversity is altered due to hormonal shift, immune and metabolic changes during this condition. Interplay of various above-mentioned factors with the microbial diversity is bidirectional in nature. During pregnancy, microbial load increases and can result in periodontal and dental problems. Various studies found a degree of link between oral polymicrobial disorders and pregnancy outcomes, but its molecular mechanism is still unclear (Borgo et al., 2014; Gonzales-marin et al., 2013; Han and Wang, 2013). Shift of oral microflora in periodontal diseases and its possible effect on PTB gained attention after work Offenbacher *et al*., (1996) in a study of pregnant females with oral health issues. However, up to now no conclusive evidence is available to support this association (Tettamanti et al., 2017; Vinturache et al., 2016; Wagle et al., 2018; Yang et al., 2015).

Previously most of the studies, which aimed to find the pregnancy associated microbial diversity and its association with oral problems and adverse pregnancy outcomes (APOs), were based on culture or PCR-based identification of periodontopathogens or cariogenic microbes in maternal oral cavity or placenta. Most of these microbes are anaerobes. Data regarding prevalence of other aerobic culturable bacterial species and also oral microbiome of postpartum females in health and disease conditions is limited. There is a need to decipher the abundance and pathogenic potential of these species in oral diseases and pregnancy outcomes. The present study was aimed to use culture-dependent and independent methods to explore salivary bacterial diversity and its pathogenic potential in postpartum phase and it's if any association with oral health issues and APOs.

## 2 Material and methods

### 2.1 Study design, settings, population, and sample collection

The present cross-sectional study was conducted after approval from ethical review boards of Capital Hospital (Islamabad), Holy Family Hospital-Rawalpindi Medical University (Rawalpindi), and Quaid-I-Azam University (Islamabad) ethical committees (QAU-BRC). In this study, postpartum females (*n* = 267) admitted in Gynaecology Department of Capital Hospital, Islamabad and Holy Family Hospital, Rawalpindi, from January 2016 to March 2018, post one day of delivery were included. Postpartum females were either having full term birth (FTB) or suffering from different APOs [PTB, Low weight birth (LWB), and preeclampsia], with or without oral health issues (gingivitis and dental caries). In addition, to postpartum females, 54 nonpregnant healthy females from general population were also included. Unstimulated saliva sample were collected from these females by passive drooling method and processed within 3 h in Molecular Medicine Laboratory, Microbiology Department, QAU, Islamabad.

Among postpartum group, samples of three females, while one from non-pregnant group was further processed for identification of salivary bacteriome by amplification of V4 region of 16S rRNA. Subjects were divided into 2 groups: 1^st^ group comprised of two postpartum female having dental caries and gingivitis, one of them (BK2) delivered pre-term low weight (PLWB) and 2^nd^ one had FTB with normal weight baby (BK1), while the 2^nd^ group comprised of healthy postpartum female without dental issues having FTB and normal weight baby (BKC1) along-with healthy non-pregnant female (BKC2).

### 2.2 Bacterial isolation by culture-based methods from saliva samples of postpartum and nonpregnant females

For isolation and purification of aerobic bacteria, nutrient agar plates (CM0003, Oxoid) were inoculated with saliva samples (0.1 mL) and incubated for 24–48 h at 37 °C. For Mutans streptococci MS (*S. mutans* and *S. sobrinus*) isolation, saliva samples were inoculated on modified sucrose bacitracin (SB-20 M) media and incubated anaerobically for 48 h at 37 °C (Saravia et al., 2013). After purification, isolated colonies were Gram stained and were subjected to standard microbiological and molecular methods for identification.

### 2.3 Bacterial DNA Extraction from saliva samples and sequencing

For bacterial DNA extraction, two-day protocol as described previously (http://www.openwetware.org/wiki/Gill:S._aureus_genomic_DNA_isolation), with few modifications was used. After DNA extraction, PCR amplification of 16S rRNA gene V4 region was carried out by using primers 515/806 having barcode on forward primer and sequencing using Illumina MiSeq plateform was at MR DNA (www.mrdnala.com, shallowater, TX, USA). Raw data sequences have been submitted in Sequence Read Archive and assigned an accession number PRJNA505914.

### 2.4 Detection of biofilm forming ability of isolated bacteria by Congo red (CRA) and microtiter plate (MTP) assay

Biofilm detection by CRA and MTP method was done by a method as described previously by Khadija *et al*., (2019). After incubation, black crusty colonies were produced by biofilm formers, while pink or red colour colonies were produced by non-biofilm producing isolates.

### 2.5 Antibacterial susceptibility testing

Antibacterial activity was checked by Kirby-Bauer disc diffusion method according to the recommendation of CLSI-2018-M100 (CLSI, 2018). All bacteria were tested for antimicrobial activity against different antibiotics and results were interpreted as recommended by CLSI-2018-M100.

### 2.6 Statistical analysis

For statistical analysis, IBM SPSS (version 21) was used. Chi-square (χ²) test and Fisher's exact test, were used to determine significant associations between different variables, where *P* < 0.05 was considered as level of significance. For risk measurements odds ratio (OR) was calculated with 95% Cl. For sequence data processing, MR DNA analysis pipeline (MR DNA, Shallowater, TX, USA) was used. Further analysis was carried out by using microbiome analyst (http://www.microbiomeanalyst.ca).

## 3 Results

### 3.1 Isolation of aerobic and facultative anaerobic bacteria from saliva samples

Out of 267 samples, 384 aerobic bacterial isolates and among nonpregnant females, out of 54 samples 69 isolates were purified. In postpartum group 174/267 (65.16%) samples, while among nonpregnant group 32/54 (59.25%) were positive for *Staphylococcus* species. Approximately one-fold increased colonization risk for *Staphylococcus* species in oral cavity of postpartum group was seen in comparison to nonpregnant females (OR = 1.28). *S. epidermidis* presence was significantly raised in postpartum females (*P* = 0.005Among postpartum group, 6.36% saliva samples were positive for *K. pneumoniae*, 5.61% *Enterobacter* species, 2.62% *E. coli*, 12.73% Gram positive rods and 10.48% for *N. meningitides*.

From postpartum group, 392 and from nonpregnant group 51 facultative anaerobic MS isolates were cultured. In postpartum group, 249 (93.26%) saliva samples were positive for *S. mutans*, while *S. Sobrinus* was positive in 143 (53.5%) females. In comparison to nonpregnant females, significantly high colonization of *S. mutans*, and an approximately nineteen-fold increased risk for colonization of *S. mutans* in postpartum females (OR = 18.64) was found (Table 1).

**Table 1:** Colonization of bacterial species in oral cavity of postpartum females (*n*=267) compared to nonpregnant females (*n*=54).

Isolates	Postpartum females *n* (%)	Nonpregnant females *n* (%)	*P*-value (χ^2^)	OR (95% Cl)
***Staphylococcus* spp.**	174 (65.16)	32 (59.25)	0.408 (0.682)	1.28 (0.70–2.34)
***S. aureus***	87 (32.58)	23 (42.59)	0.157 (1.99)	0.65 (0.35–1.18)
***S. epidermidis***	78 (29.22)	06 (11.12)	0.005 (7.61)	3.34 (1.37–8.12)
***S. saprophyticus***	32 (11.98)	02 (3.70)	0.071 (3.25)	3.54 (0.82–15.24)
***Aerobic Streptococcus* spp.**	73(27.34)	04 (7.84)	0.001 (9.78)	4.70 (1.64-13.48)
***K. pneumoniae***	17 (6.36)	04 (7.40)	0.777 (0.07)	0.85 (0.27–2.63)
***Enterobacter* spp.**	15 (5.61)	02 (3.70)	0.566 (0.32)	1.54 (0.34–6.97)
***E. coli***	07 (2.62)	01 (1.85)	0.740 (0.10)	1.42 (0.17–11.84)
**Gram positive rods**	34 (12.73)	05 (9.25)	0.475 (0.50)	1.43 (0.53–3.84)
***N. meningitides***	28 (10.48)	10 (18.52)	0.095 (2.77)	0.51 (0.23–1.13)
***S. mutans***	249 (93.26)	23 (42.60)	<0.0001 (89.1)	18.64 (9.06–38.3)
***S. sobrinus***	143 (53.5)	28 (51.85)	0.818 (0.05)	1.07 (0.59–1.92)

### 3.2 Association of bacterial species with oral disorders, dietary habits and obstetric factors

*S. aureus* colonization increased approximately one-fold risk with oral disorders in postpartum females. Its colonization was increased in postpartum females with high juice/drinks intake (OR = 0.87 to OR = 1.44). The presence of *S. epidermidis* in saliva showed approximately one-fold risk for gingivitis. Its colonization was increased in females with increasing tea/coffee intake. Among postpartum females, *S. saprophyticus* colonization increased one to two-fold risk for oral disorders and APOs. Its colonization was also increased with increasing juices/drinks intake (OR = 1.67 to OR = 2.65) (Table 2).

**Table 2:** Association of *Staphylococcus* colonization with oral disorders, dietary habits, and obstetric factors among postpartum females (*n*=267).

Factors	*S. aureus*		*S. epidermidis*		*S. saprophyticus*	
	*n* (%)	OR (95% Cl)	*n* (%)	OR (95% Cl)	*n* (%)	OR (95% Cl)
Gingivitis	25(9.36)	1.10(0.62–1.96)	23(8.62)	1.16(0.64–2.08)	09(3.37)	1.04(0.45–4.09)
Dental caries	28(10.48)	1.34(0.76–2.34)	21(7.86)	0.92(0.50–1.66)	13(4.86)	1.90(0.89–3.84)
Intake of juices/drinks No Intake Not frequently Frequently	19(7.11) 25(9.36) 43(16.10)	- 0.87(0.43–1.78) 1.44(0.79–2.62)	11(4.12) 28(10.48) 39(14.60)	- 2.12(0.96–4.68) 1.05(0.58–1.90)	03(1.12) 07(2.62) 22(8.23)	- 1.67(0.41–6.73) 2.65(1.07–6.52)
Tea and coffee intake/day No Intake 1-2 times 3-4 times >4 times daily	09(3.37) 68(25.46) 10(3.70) 00(0.00)	- 1.04(0.44–2.45) 0.63(0.29–1.38) -	07(2.62) 57(21.34) 13(4.86) 01(0.38)	- 1.15(0.46–2.88) 1.19(0.57–2.47) 2.07(0.12–35.8)	02(0.74) 25(9.36) 05(1.87) 00(0.00)	- 1.80(0.40–8.09) 0.98(0.35–2.75) -
Gestational period PTB FTB	16(5.60) 71(26.60)	0.74(0.38–1.40)	13(4.86) 65(24.34)	0.64(0.32–1.26)	09(3.37) 23(8.61)	1.48(0.64-3.41)
Baby weight (Kg) <2.5 2.5-4.0 >4	11(4.12) 72(26.96) 04(1.50)	-	13(4.86) 64(23.98) 01(0.38)	- 0.60(0.30–1.18) 1.38(0.14–13.5)	06(2.24) 24(8.98) 02(0.74)	- 0.82(0.32–2.13) 0.13(0.01–0.99)
Preeclampsia	09(3.37)	0.82(0.36–1.88)	09(3.37)	0.99(0.43–2.25)	05(1.87)	1.48(0.52–4.20)

Gingivitis and dental caries were significantly associated with *S. mutans* colonization in postpartum group (*P* = 0.007 and *P* = 0.006, respectively). Increased risk was seen for gingivitis and dental caries development in females with *S. mutans* colonization. Decreasing brushing frequency was significantly affecting the *S. mutans* colonization in oral cavity (*P* < 0.001) of these females. *S. mutans* was significantly raised in postpartum females having high juices/drinks intake (*P* = 0.01), and its colonization risk was high in females with increased tea/coffee intake (from OR = 0.40 to OR = 7.80). Although, *S. sobrinus* colonization was not significantly high in postpartum females with oral disorders, however, it showed approximately one-fold increased risk for development of gingivitis. Its colonization was significantly changed in females with low brushing frequency (*P* = 0.025) and high sugary liquid intake (juices/drinks intake *P* = 0.008 and tea/coffee intake *P* = 0.01). Like *S. mutans, S. sobrinus* also showed increased risk for colonization in postpartum females with increased intake of sugary fluids (Table 3).

**Table 3:** Associations of Mutans Streptococci (MS) with oral disorders, dietary habits, and obstetric factors among postpartum females (*n*=267).

Factors	*S. mutans*			*S. sobrinus*		
	*n* (%)	*P*-value (χ^2^)	OR (95% Cl)	*n* (%)	P-value (χ^2^)	OR (95% Cl)
Gingivitis	73(27.34)	0.007(7.26)	15.40(0.916–259.06)	40(14.98)	0.80(0.06)	1.07(0.62–1.83)
Dental caries	75(28.08)	0.006(7.54)	16.00(0.95–269.11)	37(13.86)	0.38(0.748)	0.79(0.46–1.34)
Brushing frequency/Day No brushing 1 time 2 times 3 times	04(1.50) 155(58.06) 90(33.70) 00(0.00)	<0.001(43.61)	- 2.30(0.11–46.8) 0.50(0.17–1.44) 0.01(0.0006-0.28)	03(1.12) 96(35.96) 44(16.48) 00(0.00)	0.025(9.27)	- 0.48(0.04–4.73) 0.56(3.37–0.92) 0.17(0.008–3.47)
Intake of juices/drinks No Intake Not frequently Frequently	61(22.84) 83(31.10) 105(39.32)	0.01(7.99)	- 1.12(0.006–2.27) 0.48(0.16–1.41)	31(11.61) 37(13.85) 75(28.10)	0.008(9.61)	- 0.70(0.36–1.35) 2.40(1.36–4.23)
Tea and coffee intake/day No Intake 1-2 times 3-4 times >4 times	26 (9.74) 181(67.80) 40(14.98) 02(0.74)	0.21(4.50)	- 0.40(0.05–3.20) 7.80(0.46–132.57) -	21(7.86) 96(35.96) 24(9.00) 02(0.74)	0.01(10.82)	- 0.26(0.10–0.69) 1.59(0.79–3.18) 3.36(0.15–74.74)
Gestational period PTB FTB	53(19.86) 196(73.40)	0.51(0.41)	0.70(0.23–2.06)	24 (9.00) 119(44.56)	0.03(4.41)	0.53(0.29–0.96)
Baby weight (Kg) <2.5 2.5-4.0 >4	50(18.72) 197(73.80) 02(0.75)	<0.001(25.9)	- 0.15(0.05–0.43) 0.03(0.003–0.25)	26(9.74) 115(43.08) 02(0.74)	0.189(3.32)	- 1.58(0.32–1.04) 1.30(0.18–9.46)
Preeclampsia	28(10.48)	0.48(0.48)	0.63(0.17–2.32)	05(1.88)	<0.001(19.75)	0.13(0.05–0.36)

### 3.3 Detection of biofilm forming activity expressed by oral bacteria by CRA and MTP method

Out of 210 *Staphylococcus* isolates from postpartum females 22 isolates of *S. aureus* showed positive results for biofilm formation i.e. black, dry crystalline colonies, 09 isolates of *S. epidermidis* and two *S. saprophyticus* isolates were also biofilm producers. Among nonpregnant females 11 *S. aureus* and 03 *S. epidermidis* isolates exhibited biofilm forming activity, while both *S. saprophyticus* isolates showed non-biofilm forming ability. All *K. pneumoniae* and *E. coli* isolates gave negative biofilm forming ability on CRA, while only two isolates of *Enterobacter* species from postpartum group produced black crystalline colonies on CRA plate. Among Gram positive rods, dark black dry colonies were produced by 06 isolates and among Gram negative cocci, only 09 isolates were biofilm former in postpartum group. Among nonpregnant females only one Gram-positive rod and 02 *N. meningitides* isolates exhibit biofilm forming ability.

Among all isolates, 86 (40.95%) *Staphylococcus* isolates expressed biofilm forming ability by MTP method. Out of 86 biofilm forming isolates of *Staphylococci,* only one (1.16%) isolate was strong, 03 (3.48%) moderate and 82 (95.34%) were weak biofilm formers. Greater biofilm forming ability was shown by *S. aureus* isolates (47%) followed by *S. epidermidis* (35.89%) and *S. saprophyticus* (34.37%). Among isolates from nonpregnant females, 21 (55.26%) expressed biofilm forming ability of which 17 isolates of *S. aureus*, 03 of *S. epidermidis* and only one of *S. saprophyticus* had biofilm forming activity.

All *E. coli* isolates showed weak biofilm forming ability by MTP method. Twelve of *Enterobacter* species and 10 of *K. pneumoniae* isolates were confirmed as weak biofilm formers. Among Gram-positive rods, 12 were non-biofilm formers while all remaining 64.70% were confirmed as weak biofilm formers. Eleven (39.28%) Gram-negative cocci expressed weak biofilm forming ability. Among nonpregnant group all Gram-negative rods were confirmed as non-biofilm formers while two Gram-positive and 01 Gram-negative cocci produce weak biofilms.

### 3.4 Antibiotic sensitivity testing

*S. aureus* isolates were tested against 17 antibiotics. Among cephalosporin group, 60% of the isolates showed methicillin sensitivity. Resistance was high against cefotaxime (62%). Sensitivity was very low against penicillin class with 93% and 63% resistance against penicillin and ampicillin, respectively. High resistance was also seen against quinolones (65%), antibiotics of class fluoroquinolones (45%), erythromycin (47%) and gentamycin (35%). *S. epidermidis* was tested against ten antibiotics, in which all isolates showed complete sensitivity to tetracycline, ciprofloxacin and gentamicin but chloramphenicol resistance was in 10.25% isolates. High resistance was seen against antibiotics of class penicillin and cephalosporins. *S. saprophyticus* was tested against eight antibiotics. Complete resistant to cefotaxime and penicillin was shown by all isolates. Against cefoxitin resistance was high as 65.62% (Table 4a).

**Table 4a:** Antibiotic susceptibility pattern of *Staphylococcus* species isolated from postpartum females.

Class	Antibiotics		*S. aureus n* (%)	*S. epidermidis n* (%)	*S. saprophyticus n* (%)
Cephalosporins	Cefoxitin (FOX30)	*R	40 (40.00)	47(60.26)	21(65.62)
		*S	60 (60.00)	31(39.74)	11(34.38)
	Cefotaxime (CTX30)	*R	62(62.00)	43(55.12)	32(100)
		S	38(38.00)	35(44.88)	00(0.00)
	Ceftriaxone (CRO30)	R	25(25.00)	52(66.67)	**-**
		S	70(70.00)	26(33.33)	**-**
		I	05(5.00)	0(0.00)	**-**
Penicillin	Penicillin (P10U)	R	93(93.00)	70(89.74)	32(100)
		S	07(7.00)	08(10.26)	00(0.00)
	Ampicillin (AMP10)	R	63(63.00)	35(44.87)	11(34.38)
		S	22(22.00)	35(44.87)	21(65.62)
		I	15(15.00)	08(10.26)	0(0.00)
Lincosamide	Clindamycin (DA10)	R	20(20.00)	-	-
		S	80(80.00)	-	-
Macrolides	Erythromycin (E15)	R	47(47.00)	26(33.33)	11(34.38)
		S	22(22.00)	44(56.41)	00(0.00)
		I	31(31.00)	08(10.26)	21(65.62)
Tetracycline	Tetracycline (TE30)	R	25(25.00)	00(0.00)	-
		S	60(60.00)	78(100)	-
		I	15(15.00)	00(0.00)	-
Linezolid	Linezolid (LZD10)	R	15(15.00)	-	-
		S	85(85.00)	-	-
Fluoroquinolones	Ofloxacin (OFX5)	R	45(45.00)	-	-
		S	45(45.00)	-	-
		I	10(10.00)	-	-
	Ciprofloxacin (CIPS5)	R	45(45.00)	00(0.00)	-
		S	55(55.00)	78(100)	-
		I	00(0.00)	00(0.00)	-
Chloramphenicol	Chloramphenicol (C30)	R	08(8.00)	08(10.25)	00(0.00)
		S	69(69.00)	52(66.68)	11(34.38)
		I	23(23.00)	18(23.07)	21(65.62)
Nitrofurantoin	Nitrofurantoin (F300)	R	10(10.00)	-	00(0.00)
		S	90(90.00)	-	32(100)
Sulfonamides	Sulphamethox/ Trimethoprim (SXT25)	R	15(15.00)	-	**-**
		S	85(85.00)	-	**-**
		I	00(0.00)	-	**-**
Quinolones	Nalidixic acid (NA30)	R	65(65.00)	-	11(34.38)
		S	25(25.00)	-	21(65.62)
		I	10(10.00)	-	00(0.00)
Aminoglycosides	Amikacin (AK30)	R	05(5.00)	-	-
		S	80(80.00)	-	-
		I	15(15.00)	-	-
	Gentamicin (CN10)	R	35(35.00)	00(0.00)	-
		S	65(65.00)	78(100)	-
		I	00(0.00)	00(0.00)	-

*K. pneumoniae* and *E. coli* isolates were tested against 18 antibiotics. Isolates of *K. pneumoniae* showed complete resistance against most of the antibiotics such as erythromycin and penicillin. Comparatively high resistance was also seen against ampicillin, clindamycin, tobramycin, tetracycline (88.24% against each), cephalosporins (82.36% against cefotaxime and 47.06% against ceftriaxone), linezolid (64.70%), nitrofurantoin (52.94%), chloramphenicol and nalidixic acid. All *E. coli* isolates were 100% resistant to ceftriaxone, penicillin, clindamycin, erythromycin, tetracycline and linezolid. But 71.42% isolates were resistant to nalidixic acid, nitrofurantoin and ciprofloxacin. *Enterobacter* species were tested against ten antibiotics. Their sensitivity against most of the antibiotics was 100% like the antibiotics of class aminoglycosides and carbapenems followed by 86.66% against chloramphenicol, 80% against ofloxacin, 66.67% against cefotaxime, 46.66% against each ceftriaxone and ampicillin (Table 4b).

**Table 4b:** Antibiotic sensitivity pattern of species of Enterobacteriaceae members isolated from saliva of postpartum females.

Class	Antibiotics		*K. pneumonia n* (%)	*E. coli n* (%)	*Enterobacter* species *n* (%)
Cephalosporins	Cefotaxime (CTX30)	R	14(82.36)	02(28.75)	05(33.33)
		S	03(17.64)	05(71.42)	15(66.67)
	Ceftriaxone (CRO30)	R	08(47.06)	07(100)	08(53.34)
		S	09(52.94)	00(0.00)	07(46.66)
		I	00(0.00)	00(0.00)	00(0.00)
Penicillin	Penicillin (P10U)	R	17(100)	07(100)	-
		S	00(0.00)	00(0.00)	-
	Ampicillin (AMP10)	R	15(88.24)	02(28.75)	06(40.00)
		S	02(11.76)	00(0.00)	07(46.66)
		I	00(0.00)	05(71.42)	02(13.34)
Lincosamide	Clindamycin (DA10)	R	15(88.24)	07(100)	-
		S	02(11.76)	00(0.00)	-
Macrolides	Erythromycin (E15)	R	17(100)	07(100)	-
		S	00(0.00)	00(0.00)	-
		I	00(0.00)	00(0.00)	-
Tetracycline	Tetracycline (TE30)	R	15(88.24)	17(100)	03(20.00)
		S	02(11.76)	00(0.00)	12(80.00)
		I	00(0.00)	00(0.00)	00(0.00)
Linezolid	Linezolid (LZD10)	R	11(64.70)	17(100)	-
		S	06(35.30)	00(0.00)	-
Fluoroquinolones	Ofloxacin (OFX5)	R	02(11.76)	00(0.00)	00(0.00)
		S	15(88.24)	05(71.42)	12(80.00)
		I	00(0.00)	02(28.75)	03(20.00)
	Ciprofloxacin (CIPS5)	R	03(17.64)	05(71.42)	-
		S	11(64.70)	00(0.00)	-
		I	03(17.64)	02(28.75)	-
Chloramphenicol	Chloramphenicol (C30)	R	08(47.06)	02(28.75)	02(13.34)
		S	09(52.94)	05(71.42)	13(86.66)
		I	00(0.00)	00(0.00)	00(0.00)
Nitrofurantoin	Nitrofurantoin (F300)	R	09(52.94)	05(71.42)	-
		S	08(47.06)	02(28.75)	-
Sulfonamides	Sulphamethox/ Trimethoprim (SXT25)	R	02(11.76)	00(0.00)	-
		S	15(88.24)	17(100)	-
		I	00(0.00)	00(0.00)	-
Quinolones	Nalidixic acid (NA30)	R	08(47.06)	05(71.42)	-
		S	09(52.94)	02(28.75)	-
		I	00(0.00)	00(0.00)	-
Aminoglycosides	Tobramycin (TOB30)	R	15(88.24)	00(0.00)	00(0.00)
		S	02(11.76)	17(100)	15(100)
	Amikacin (AK30)	R	00(0.00)	00(0.00)	00(0.00)
		S	17(100)	17(100)	15(100)
		I	00(0.00)	00(0.00)	00(0.00)
	Gentamicin (CN10)	R	05(29.42)	02(28.75)	00(0.00)
		S	12(70.58)	05(71.42)	15(100)
Carbapenems	Imipenem IMI10	R	00(0.00)	00(0.00)	00(0.00)
		S	17(100)	17(100)	15(100)

*R: Resistant, *S: sensitive, *I: Intermediate

### 3.5 Characteristics of sequenced bacterial DNA samples

A total of 6,467,93 reads were obtained from four saliva samples. BK1 had total count of 1,349,91, BK2 1,783,28, BKC1 had 1,563,84, and BKC2 had 1,770,90. A total of 527 OTUs were detected with sequence similarity ≥ 97% in these samples. Microbiome analyst tool using low count filter, OTUs with less than 20% prevalence in sample were removed. After data trimming, filtering and normalization, 420 OTUs were predominant with count ≥2.

#### 3.5.1 Taxonomic analysis of sequenced data

A total of 16 phyla, 25 classes,43 orders, 84 families, 156 genera and 282 species were observed in all samples. Subject BK1 had 16 phyla, dominated by proteobacteria (47.06%) followed by Firmicutes (31.55%), Bacteroidetes (9.16%), Fusobacteria (6.57%), Actinobacteria (3.91%), Spirochaetes (1.3%), Tenericutes (0.34%), Cyanobacteria (0.062%), Planctomycetes (0.026%), Acidobacteria (0.0098%), Synergistetes (0.0065%), Deferribacteres (0.0065%), Deinococcus Thermus (0.0065%), Gemmatimonadetes (0.004%) and Candidatus saccharibacteria (0.0008%). Subject BK2 had 12 phyla, dominated by Firmicutes (64.24%) followed by Proteobacteria (12.03%), Bacteroidetes (10.86%), Actinobacteria (10.11%), Fusobacteria (2.55%), Spirochaetes (0.14%), Tenericutes (0.03%), Acidobacteria (0.003%), Candidatus saccharibacteria (0.0008%), Synergistetes (0.0019%), Deferribacteres (0.0019%) and Cyanobacteria (0.0013%). In case of BKC1 there were 12 phyla, which was again dominated by Firmicutes (50.28%) followed by Bacteroidetes (21.69%) Proteobacteria (11.15%), Actinobacteria (9.66%), Fusobacteria (6.41%), Cyanobacteria (0.57%), Spirochaetes (0.157%), Tenericutes (0.021%), Candidatus saccharibacteria (0.012%), Synergistetes (0.011%), Acidobacteria (0.0014%), and Deferribacteres (0.00072%). Subject BKC2 had 13 phyla, dominated by Firmicutes (41.29%) followed by Proteobacteria (35.07%), Bacteroidetes (7.50%), Fusobacteria (7.40%), Actinobacteria (7.10%), Spirochaetes (1.46%), Tenericutes (0.049%), Acidobacteria (0.046%), Synergistetes (0.031%), Deferribacteres (0.022%), Cyanobacteria (0.018%), Candidatus saccharibacteria (0.004%), and Deinococcus Thermus (0.0006%).

In these samples, abundance of major genera were recorded. In BK1 major genera were: *Streptococcus* (20.91%) followed by *Yersinia* (20.76%), *Haemophilus* (5.90%), *Neisseria* (5.90%), *Fusobacterium* (4.58%), *Gemella* (3.90%), *Prevotella* (3.64%), *Aggregatibacter* (3.14%), *Rothia* (2.54%), *Agrobacterium* (2.49%), *Veillonella* (2.35%) and *Porphyromonas* (2.29%). In BK2 dominant genera were: *Streptococcus* (40.75%), followed by *Gemella* (12.47%), *Prevotella* (9.69%), *Rothia* (6.55%), *Veillonella* (4.29%), *Haemophilus* (4.24%), Neisseria (4.086%), *Granulicatella* (4.082%) and *Actinomyces* (2.911%). BKC1 had predominant genera including *Streptococcus* (30.04%) followed by *Prevotella* (13.35%), *Gemella* (6.61%), *Granulicatella* (4.51%), *Rothia* (4.09%), *Actinomyces* (3.68%), *Fusobacterium* (3.36%), *Porphyromonas* (3.26%), *Neisseria* (3.07%), *Leptotrichia* (2.98%), *Chryseobacterium* (2.76%), *Lautropia* (2.66%) and *Abiotrophia* (2.20%). In BKC2, most abundant genera were: *Streptococcus* (26.05%), followed by *Neisseria* (21.64%), *Haemophilus* (8.05%), *Fusobacterium* (5.88%), *Rothia* (5.67%), *Gemella* (4.66%), *Granulicatella* (4.16%), *Prevotella* (3.45%), *Porphyromonas* (3.43%) and *Aggregatibacter* (2.98%).

#### 3.5.2 Community profiling

##### 3.5.2.1 Alpha and beta diversity

Alpha diversity was assessed by Shannon index, Simpson's index and Chao 1. Alpha diversity was high in females with FTB compared to PLWB. Shannon index for BK1, BKC1 and BKC2 saliva samples showed relatively high richness and evenness compared to BK2 sample, which have PLWB. Although both BK1 and BK2 postpartum females were suffering from gingivitis and dental caries, but BK1 delivered a normal weight full term baby. Results of Simpson diversity index also showed greater diversity in BK1, BKC1 and BKC2 compared to BK2. However statistical analysis by Mann-Whitney/Kruskal-Wallis method showed insignificant results between samples (Table 5). Bray-Curtis dissimilarity showed greater dissimilarity between BK1 and BK2 sample (BC = 0.55) compared to BK1 and BKC1 (BC = 0.54), BK1 and BKC2 (BC = 0.52), and BKC1 and BKC2 (BC = 0.43).

**Table 5:** Alpha diversity indices for salivary microbiome of postpartum and nonpregnant females.

Sample ID	Shannon Index	Simpson's Index	Chao 1 index
BK1	3.50	0.916	385.06
BK2	3.05	0.858	389.66
BKC1	3.61	0.914	388.75
BKC2	3.38	0.907	386.73

##### 3.5.2.2 Core microbiome analysis

At genera level, 85 out of 156 genera were shared between all four female saliva samples. Firmicutes, Proteobacteria, Bacteroidetes, Fusobacteria, Actinobacteria were the top taxa shared by all females. At genera level *Streptococcus, Veillonella, Prevotella, Neisseria, Gemella, Rothia, Haemophilus, Fusobacterium, Porphyromonas, Granulicatella, Leptotrichia, Campylobacter, Actinomyces, Aggregatibacter, Lautropia,* and *Eubacterium* were top genera.

## 4 Discussion

Oral cavity comprised of an open, diverse and dynamic system, which is heterogeneous in nature harbouring both exo- and endogenous symbiotic microbial species in different oral sites to maintain oral healthy environment(Zawadzki et al., 2017; Zawadzki et al., 2016). This environment can change to pathogen rich ecosystem due to several nutritional, metabolic, immune, and structural alterations. Inflammatory immune response initiated against such colonization can lead to destruction of gingival and tooth surfaces causing periodontal diseases and caries lesions literature showed that these microbes in oral cavity, their toxins and by-products can enter the systemic circulation from localized asymptomatic lesions and contribute to the production of various systemic disorders such as pulmonary disorders, cardiovascular disorders and adverse pregnancy outcomes (APOs) (Ballini et al., 2020; Ballini et al., 2020; Kumar, 2017; Inchingolo et al., 2020; Isacco et al., 2021).

In present study, salivary microbiota was analysed in postpartum females and was compared with healthy nonpregnant females. Furthermore, its association with oral disorders and APOs was also assessed. Differences in species prevalence between both groups of females were found. Frequently detected species in both healthy and postpartum females by culture-based method, belonged to *Staphylococcus, Streptococcus*, Enterobacteriaceae*, Neisseria* and Gram-positive rods. Prevalent species were *S. mutans, S. sobrinus, S. aureus, S. epidermidis, S. saprophyticus, E. coli and K. pneumoniae,* cultured from these females.

*Staphylococcus* species were the most prevalent aerobic bacterial species in oral cavity of postpartum compared to nonpregnant females. The isolation rate of *Staphylococcus* species differed in both groups showed clearly, shift in Staphylococcal species diversity especially more pronounced in postpartum females. *Staphylococcus* species were believed as transient oral flora. It was present in both type of females strengthens the finding that it is a common resident of healthy oral cavity especially in saliva, tongue and supragingival plaque. Its oral carriage is reported from 61.36%–94.0% in oral cavity, which is in line with our work on Pakistani females where its carriage rate was 65.16%(Jackson et al., 1999; Loberto et al., 2004; Ohara-Nemoto et al., 2008).

Despite having pathogenic potential for nosocomial infection, *Staphylococcus* species are not frequently studied for their role in oral disorders (Loberto et al., 2004). Compared to present work, comparable results about the isolation of *Staphylococcus* species in patients suffering from gingivitis (8%) were reported by Koukos et al., (2015). In present study, 10.48% postpartum females suffering from dental caries were positive for *S. aureus* isolation. This rate was lower than reported from a study conducted on caries active supragingival plaque samples (16%), however, colonization was non-significant in subjects of both studies (Hoceini et al., 2016). Methicillin resistance was high in isolates of present study from postpartum females. Their resistance frequency was high from previous reports (Cruz et al., 2011; McCormack et al., 2015).

Daniluk *et al*., (2006) conducted a study on isolation of oral aerobic bacterial species in denture wearing and non-wearing diabetic and cancer patients. From these dentures Gram-negative cocci were isolated, but compared to present work in which 10.48% in postpartum and 8.52% in nonpregnant females detected, a high frequency of *Neisseria* Species were detected in denture of cancer (25%) and diabetic patients (57.9%). Also, isolation frequency of *E. coli* (6.3% in cancer and in diabetic 15.8%), and *K. pneumoniae* was high in comparison to present work. Both cancer and diabetes are conditions where immunity is dampened similar to postpartum stage, where there is compromised immunity, but higher rate of isolation could be accounted for by the type of sample source which was denture which serve as conducive site for colonization and biofilm development.

Postpartum females studied in the present work showed high prevalence of *S. mutans* (93.2%) and *S. sobrinus* (53.5%) in postpartum females, compared to non-pregnant group (42.6% and 51.85%, respectively). After *S. mutans, S. sobrinus* is also considered as an important cariogenic pathogen (Nurelhuda et al., 2010). In the past, MS colonization and oral disorders association have been only seen in children Ito (2000). No study is available in Pakistani population on the prevalence of MS in postpartum females, even pregnant females are rarely investigated. Similar to present work, a study from neighbouring country India reported significantly high *S. mutans* colonization in oral cavity of females of postpartum phase compared to nonpregnant females (Kamate et al., 2017). Beside high MS colonization in present study females, a significantly higher salivary *S. mutans* prevalence was found in postpartum females suffering from dental caries (*P* = 0.006) and gingivitis (*P* = 0.007). It is now becoming well established fact from several reports that acidic environment during pregnancy promotes the growth of these cariogenic microbes and increase risk for tooth demineralization (Marla et al., 2018; Silk et al., 2008).

Most of the salivary isolates of postpartum females were expressing biofilm forming ability and high antibiotic susceptibility profile. In oral cavity, bacteria mostly lived as a part of biofilms. In comparison to planktonic form, biofilm associated bacteria showed more resistant to antibiotics (Pinheiro et al., 2014; Shrestha et al., 2018).

In present study unculturable bacterial diversity was also analysed by using NGS technique. There is a scarcity of data related to oral microbiome in postpartum female with dental issues and its association with pregnancy outcomes. To the best of our knowledge the only study related to oral microbiome of postpartum female was conducted by Balan *et al.,* in 2018. They collected the saliva sample and supragingival plaque (SGP) samples from healthy pregnant female during each trimester and in postpartum period, showed that during pregnancy, oral microbiome shows pathogenic shift and it reverts to normal in postpartum period. However, they did not specify the time of collection of saliva sample in postpartum period and these females were healthy in terms of local oral and systematic conditions. According to this study, alpha diversity of microbiome in saliva sample of pregnant females does not change dramatically. Predominant phyla during pregnancy were Firmicutes followed by Bacteroidetes and Actinobacteria, in both saliva and SGP samples (Balan et al., 2018).

In present study, salivary microbiome of postpartum showed similar trend as reported by Balan in pregnant females in terms of core predominant phyla and genera shared by all females. This similarity of postpartum microbiome with pregnancy associated microbiome can be due to the fact, that microbiome return to its normal health associated conditions after few weeks in postpartum period, and in the present study, saliva samples was collected only after first day of delivery, that's why it was more like pregnancy associated microbiome. As the pregnancy proceeded towards the end, oral cavity is dominated by pathogenic gingivitis and dental caries associated agents (Bieri et al., 2013).

A study conducted by Anukam *at el*., in 2017 on oral microbiome of healthy premenopausal Nigerian female by using MiSeq Illumine platform showed that Firmicutes were the most abundant phyla in this group, followed by Proteobacteria, Actniobacteria, Bacteroidetes and Fusobacteria. Most abundant species which were detected include *H. parainflunzae* (80.65%) followed by *H. influenzae* (4.13%), *A. segnis* (2.96%), *Actinobacillus porcinus* (1.64%), *Veillonella spp.* (1.52%), *Lautotropia spp. TeTO* (1.36%) and *R. dentocariosa* (0.95%) (Anukam and Agbakoba, 2017). Although in present study same trend was seen in term of core microbiome phyla and core genera with healthy individuals, however, their abundance was varied between different samples, this change was since pregnancy is itself a factor that influences the microbial composition in various body sites and every second in three females suffer from dental problems.

Subject BK1 had dental problem and give full term birth showed more alpha diversity compared to BK2. Although, both BK1 and BK2 were the cases of dental problems, but their pregnancy outcomes were different. It could be due to different diversity pattern and microbial abundance between these two samples. It is also well-known fact that diversity decreases with dental problems, that is why, there might be possibility of link between decreasing bacterial diversity in females with APOs. In addition to alpha diversity, dissimilarity value between BK1 and BK2 was also high. Subject BKC1 who had good oral health and delivered FTB also showed greater diversity compared to BK2 as their diversity index was closer to BK1. This further support the finding that high microbiome diversity closer to healthy microbiome might be helpful in uncomplicated delivery.

## 5 Conclusion

The findings from the present work provides a new set of information about the salivary bacterial carriage in the early postpartum phase. In this phase females are more susceptible for colonization of pathogenic microbes expressing biofilm forming ability and increased antibiotic resistance. The limitation of the present study was that only focus was on salivary microbiota, analysis of other side in addition with saliva can be prove useful in understanding the overall picture for the effects of changing microbial composition in multiple oral sites for development of oral, dental and systematic abnormalities leading to APOs. Moreover, due to small sized study population for NGS analysis, conclusive diversity cannot be proposed as there is inter individual variation in microbiome composition. Studies regarding the pathogenic potential of oral culturable bacteria in developing dental caries, periodontal disorders and causing APOs are limited during pregnancy and postpartum phase. This present work attempts to find association of microbes with oral disorders and its possible association with APOs.

## Author Contributions

Conceptualization, methodology, project administration, visualization, validation, writing-original draft was done by B.K, R.F, S.H; software, formal analysis, resources, investigation, writing- review and editing, Data curation was done by B.K, L.B, A.S, B.R, A.S; funding acquisition was done by R.F, B,K; Supervision was done by R.F, S.H. All authors have read and agreed to the present version of the manuscript.

## Declaration of Competing Interest

The authors declare that they have no known competing financial interests or personal relationships that could have appeared to influence the work reported in this paper.
